# To clean or not to clean: Cleaning mutualism breakdown in a tidal environment

**DOI:** 10.1002/ece3.6120

**Published:** 2020-02-28

**Authors:** Katie Dunkley, Ashley J. W. Ward, Sarah E. Perkins, Jo Cable

**Affiliations:** ^1^ School of Biosciences Cardiff University Cardiff UK; ^2^ School of Biological Sciences The University of Sydney Sydney NSW Australia

**Keywords:** cleaner fish, context‐dependency, Great Barrier Reef, *Labroides dimidiatus*, mutualism, parasitism, symbiosis

## Abstract

The dynamics and prevalence of mutualistic interactions, which are responsible for the maintenance and structuring of all ecological communities, are vulnerable to changes in abiotic and biotic environmental conditions. Mutualistic outcomes can quickly shift from cooperation to conflict, but it unclear how resilient and stable mutualistic outcomes are to more variable conditions. Tidally controlled coral atoll lagoons that experience extreme diurnal environmental shifts thus provide a model from which to test plasticity in mutualistic behavior of dedicated (formerly obligate) cleaner fish, which acquire all their food resources through client interactions. Here, we investigated cleaning patterns of a model cleaner fish species, the bluestreak wrasse (*Labroides dimidiatus*), in an isolated tidal lagoon on the Great Barrier Reef. Under tidally restricted conditions, uniquely both adults and juveniles were part‐time facultative cleaners, pecking on *Isopora palifera* coral. The mutualism was not completely abandoned, with adults also wandering across the reef in search of clients, rather than waiting at fixed site cleaning stations, a behavior not yet observed at any other reef. Contrary to well‐established patterns for this cleaner, juveniles appeared to exploit the system, by biting (“cheating”) their clients more frequently than adults. We show for the first time, that within this variable tidal environment, where mutualistic cleaning might not represent a stable food source, the prevalence and dynamics of this mutualism may be breaking down (through increased cheating and partial abandonment). Environmental variability could thus reduce the pervasiveness of mutualisms within our ecosystems, ultimately reducing the stability of the system.

## Graphical abstract

In a tidal lagoon in the Great Barrier Reef, we uniquely show that both adult and juvenile bluestreak cleaner wrasse (*Labroides dimidiatus*) are part‐time facultative cleaners also pecking on the coral *Isopora palifera* for food acquisition. We also show that, contrary to well‐established patterns for this cleaner species, juveniles bite (“cheat”) their clients more than adults, exploiting mutualistic cleaning. Overall, in this tidal environment, we document a partial breakdown in the prevalence and dynamics of this iconic mutualistic cleaning interaction.

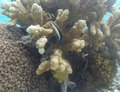

## 1 INTRODUCTION

Mutualisms, which involve beneficial interactions between species, are highly context‐dependent interactions (Chamberlain, Bronstein, & Rudgers, 2014), with their outcomes varying both temporally and spatially (Bronstein, 1994). Most mutualisms are service–resource interactions whereby one member gains food resources (e.g., nectar or ectoparasites), while the other receives a beneficial act (pollination, Landry, 2012, or parasite removal, Grutter, 1996). Differing contexts will influence the magnitude of benefits each interacting partner gains, facilitating how they behave (Frederickson, 2017). As such, under certain contexts, partners may benefit more if they exploit (e.g., cheat) or abandon the other (Sachs, Mueller, Wilcox, & Bull, 2004). Indeed, mutualistic interactions are not immune to breakdowns (e.g., a shift to parasitism, Irwin, Bronstein, Manson, & Richardson, 2010, and abandonment, Pellmyr, Leebens‐Mack, & Huth, 1996, Werner et al., 2018), which can be defined as the loss of cooperative phenotypes in a mutualist's lineage over time (Sachs & Simms, 2006). It is thus unknown how stable mutualisms really are to fluctuating environmental contexts.

Aside from plant–pollinator interactions, cleaner‐client mutualisms are the most referenced service–resource mutualisms and are ubiquitously observed on coral reefs (White, Grigsby, & Warner, 2007). For dedicated (formerly termed obligate) full‐time cleaners, there is an absolute reliance on client‐gleaned material for food (Vaughan, Grutter, Costello, & Hutson, 2017). This behavior is hence considered a specialist foraging strategy (Dunkley, Cable, & Perkins, 2018) which will only be adaptive when there is a plentiful and stable supply of food (West‐Eberhard, 1989). As a result, exploitation, through cheating (removal of material that is detrimental to the clients’ health, but beneficial to the cleaner; Poulin & Vickery, 1995), is commonplace among dedicated cleaners (Vaughan et al., 2017). However, evidence is lacking that these interactions are ever abandoned and/or fully breakdown (Frederickson, 2017; Sachs & Simms, 2006).

Mutualistic breakdowns may be facilitated by phenotypic plasticity, in other words the tendency of a genotype to produce different phenotypes under asymmetric environments (Thibert‐Plante & Hendry, 2011). Plasticity allows animals to adjust their traits to their immediate environment, increasing their fitness (van Buskirk, 2012). Many species can adapt their foraging behaviors, for example, in response to rapid environmental changes (Gilmour et al., 2018). Phenotypic plasticity is considered to be especially prevalent in variable environments where conditions can change over relatively short periods of time or are spatially patchy (Snell‐Rood, 2013). It is well established that fluctuating tidal environments promote local behavioral adaptions (Gibson, 1986; Reese, 1969) due to regular spatiotemporal changes in food availability, predator abundance (Gibson et al., 1998; McIvor & Odum, 1988) and altered physiological conditions (e.g., elevated temperature, low pH, and low dissolved oxygen, Silverman et al., 2012). Thus, these tidal systems provide a unique platform to examine phenotypic changes and adaptive shifts in mutualistic interactions. The dedicated Indo‐Pacific cleaner species, the bluestreak wrasse (*Labroides dimidiatus*), is a model species for cleaner–client interaction studies and has been shown to adjust its behavioral interactions with clients under altered physiological conditions ex situ (Paula et al., 2019) and client densities *in* and ex situ (Triki, Wismer, Levorato, & Bshary, 2018). These widely prevalent cleaners (Vaughan et al., 2017) are found across reef types but no study has yet documented the dynamics of their cleaning interactions within strongly tidal environments: flexibility in cleaning behavior is expected.

In this study, we observed the cleaning behaviors and habitat use of the bluestreak wrasse within a tidally controlled lagoon on the Great Barrier Reef to investigate whether cleaning will function adaptively under tidal environmental conditions. Tidal lagoons, found within coral reef atolls, can be isolated from the surrounding ocean for periods of the day restricting fish movement (Thresher, 1983), and hence ectoparasite abundance and diversity (Grutter, 1994), and altering their physiological conditions (Silverman et al., 2012). In this study, we compared the behaviors of adult and juvenile wrasse, to determine whether adults show plasticity in their mutualistic behavior. Cleaning foraging behavior is closely linked to their habitat use (Mills & Côté, 2010; Oates, Manica, & Bshary, 2010); thus, we first asked whether juveniles and adults differ in the use of their cleaning stations (topological features of the environment; Potts, 1973). Within this tidal environment, bluestreak wrasse have already been anecdotally observed to “wander” across the reef away from their cleaning stations (Wilson, Krause, Herbert‐Read, & Ward, 2014). Thus, here we quantified this wandering behavior for the first time and investigated a potential function, to seek out food either mutualistically (through cleaning) or through parasitic exploitation (biting client skin, cheating; Cheney & Côté, 2005). Bluestreak wrasse usually balance their mutualistic versus parasitic cleaning behaviors to ensure the mutualism is maintained (Binning et al., 2017; Bshary, 2002), but in this tidal environment, where food availability may be tempo‐spatially limited, we expected cleaners to exploit their clients more (i.e., high biting frequency with low cleaning frequency and duration), breaking down the mutualism. We did not hypothesize to observe a mutualism abandonment, as observed for other mutualists (Sachs & Simms, 2006), as bluestreak wrasse have not been previously documented to abandon cleaning all together, even under home aquarium conditions.

## 2 MATERIALS AND METHODS

### 2.1 Tidal reef environment

**Figure 1:**
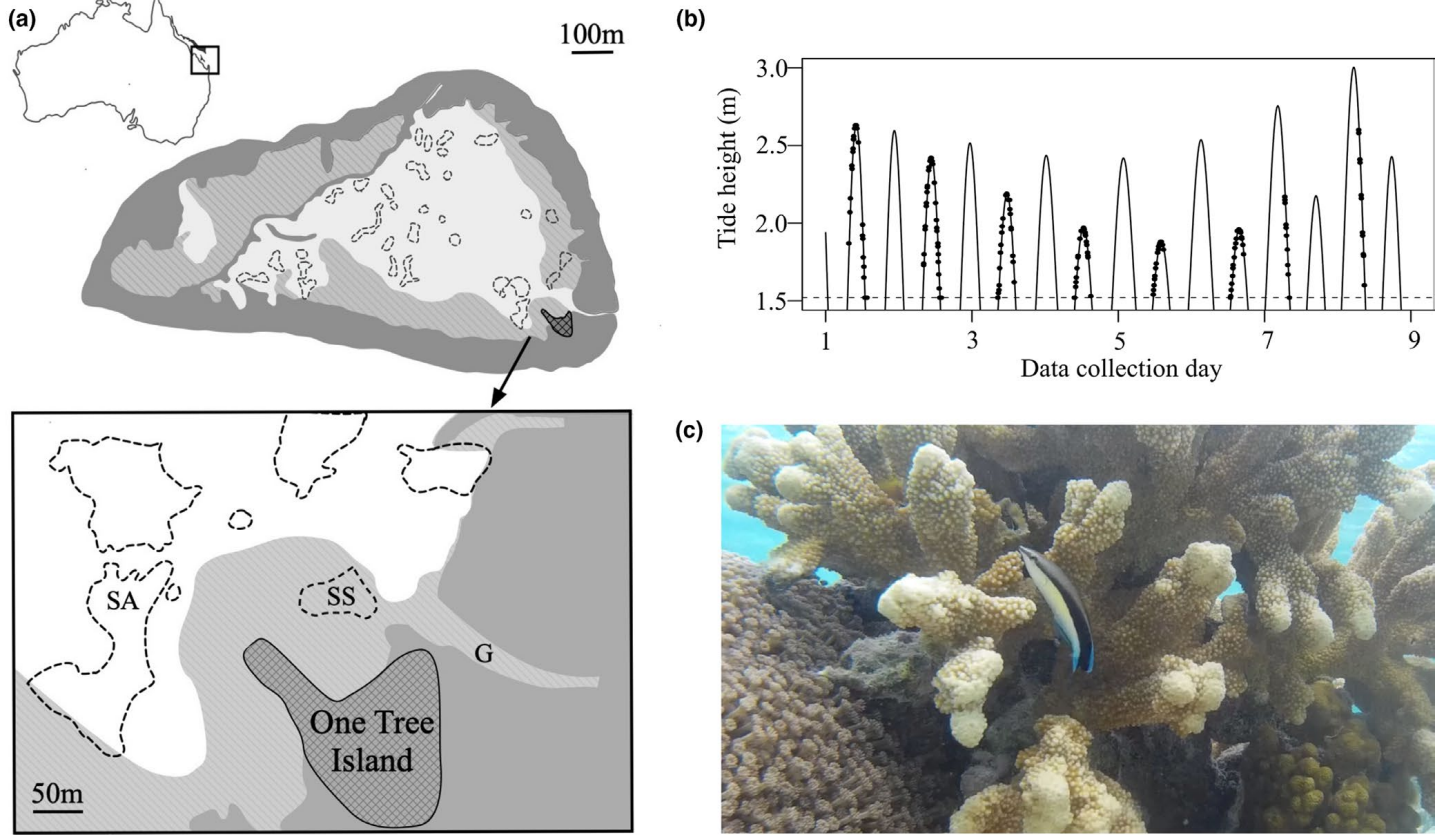
(a) Bluestreak wrasse (*Labroides dimidiatus*) study was conducted within the first lagoon of One Tree Reef, Great Barrier Reef. The work took place at SS (study site) which was close to the gutter (G). Shark Alley (SA) has been added for reference. One Tree Island is the base of the University of Sydney's Research Station. Light gray area represents sandy substrate, while darker gray represents atoll edges and barriers to the surrounding ocean. (b) Diurnal tidal range over which the study took place. Points indicate observation time‐points. Dotted line represents minimum tide height of 1.52 m. (c) Bluestreak wrasse associating with the coral *Isopora palifera*. *Isopora palifera* is a common shallow water reef building branching coral that dominates low energy lagoon environments (Ayre, Veron, & Dufty, 1991; Benzie, Haskell, & Lehman, 1995). *Isopora* coral is at high risk from bleaching induced extinction (Muir, Marshall, Abdulla, & Aguirre, 2017)

Bluestreak cleaner wrasse (*L. dimidiatus*) habitat use and cleaning behavior were observed in March 2018 on One Tree Reef (23°30′S, 152°06′E) situated in the Capricornia Cays National Park in the Southern Great Barrier Reef (Figure 1a). One Tree Reef is characterized by three shallow lagoons with high and unbroken reef crests, such that the first lagoon (Figure 1a) is isolated from the surrounding ocean for half of each tidal cycle. Due to this isolation, the tide within this first lagoon never drops below 1.52 m (Ludington, 1979, Figure 1b).

The study was conducted in this first lagoon (depth 0.5–2.5 m) on cleaners occupying shallow coral bommies on patch reef close to the coral cay, One Tree Island (Figure 1a). Low tide residual drainage from the lagoon meant that reef peaks could, at times, be above the water surface (Kinsey, 1978; Ludington, 1979). Behavioral observations (*n* = 179) conducted by snorkeling in daylight, between 6 a.m. and 6 p.m., mostly occurred when the tide was flowing (Figure 1b). The reef area was predominantly characterized by sand (mean ± *SE* proportion of 12 × 30 m benthic transects: 40.2% ± 5.42) and hard corals (32.4% ± 4.36). As a result of tidal changes, the salinity and temperature of the study environment would have also fluctuated (Silverman et al., 2012).

### 2.2 Cleaner habitat use in a tidal environment

Adult and juvenile bluestreak cleaner wrasse are morphologically distinct: adults are identified by blue and yellow coloration separated by a longitudinal black line (fork length: 4–10 cm), while juveniles are characterized by a black body and blue dorsal line (fork length: 2–3 cm; Potts, 1973). Adult and juvenile cleaners maintain separate cleaning stations which were identified by repeatedly observing the locality of individuals on the reef in the 2 days preceding data collection. Since cleaners occupied shallow coral bommies as cleaning stations, the boundaries of their isolated reef structures were clear to observers. Cleaning station locations were numbered and marked (adult *n* = 6 and juvenile *n* = 13) and the depth, height and width of each station were measured (to the nearest cm) to calculate ellipsoid station volumes (after Adam, 2011). When a cleaner occupied a station, which consisted of two or more closely isolated reef structures, ellipsoid volumes were calculated separately for each and summed. Within the study site (SS, Figure 1a), we marked all adult stations, but it was not possible to mark all juvenile stations due to the high number of individuals, and the frequent appearance of new stations. Adults can share their station with another adult (observed on five out of six of our stations) usually of the opposite sex (Robertson, 1972), while juveniles are often observed sharing their stations with “wigglers”—the smallest recognized size category of bluestreak wrasse (mean fork length: 10 mm; Potts, 1973). The behavior and presence of wigglers was not recorded due to their size and tendency to seek cover making observations challenging. The repeated measures of cleaner behavior from the same station likely represent the same individual for juveniles, and one of two individuals for adults (it was not possible to identify each different individual). For adults, where two wrasse occupied the station, we ensured that we observed both individuals across repeated observations using size differentiation: this aimed to account for sex differences in cleaning behavior (Gingins & Bshary, 2014). All observers (*n* = 3) were aware about the potential presence of cleaner fish mimics (*Aspidontus taeniatus* and *Plagiotremus rhinorhynchos*; Cheney & Marshall, 2009) on the reef and can confirm the correct species identification of focal wrasse.

Cleaners did not spend all their time at their cleaning stations, and like Wilson et al. (2014) who also observed bluestreak wrasse on One Tree Reef, we found that some fish travelled (hereafter termed “wandering,” see Dunkley et al., 2018) considerable distances from their cleaning stations (>20 m). Within the 20‐min observations, we recorded the time cleaners spent at their station versus time spent wandering. Cleaners were also observed visiting other marked stations but given that we did not mark all juvenile stations on the reef, and this behavior was uncommon (observed in 16.8% of observations, accounting for 3.8% of total observation time), we combined this behavior with wandering.

### 2.3 Cleaner behavior in a tidal environment

Both adult and juveniles were observed for up to 20 min or until the fish was lost (adults: 64 observations totaling 17 hr, 53 min, and 18 s; juveniles: 115 observations totaling 34 hr, 56 min, and 32 s; minimum observation time included in analyses: 105 s). Fish observations began at marked cleaning stations (*n* = 8.52 ± 0.71, mean ± *SE*, observations per cleaning station). We confirmed that all cleaners did associate with a cleaning station, by following cleaners ad hoc wandering on the reef, when not recording, to ensure there was no bias in the results. All behaviors were recorded by one of three snorkeling observers whom maintained a distance of 1.5 m from the cleaner.

During each observation, we recorded any cleaning interactions (defined as visible pecks on client body, as in Dunkley et al., 2018): the number of cleaning events, the time a cleaner spent associating with each client during cleaning, and the occurrence of cheating (biting of client skin or mucus identified through client jolting; Bshary & Grutter, 2002). Cleaners could also interact with clients without cleaning, either through inspections (associating with clients but not pecking body) or by chasing (swimming after clients), so we also recorded the combined frequency of these noncleaning client interactions (like Dunkley et al., 2018). Since, bluestreak wrasse cleaning behavior can be affected by the presence of a conspecific (Gingins & Bshary, 2014), we additionally recorded the time focal cleaners spent with conspecifics. All behaviors were separated as to whether they occurred at the cleaning station or when wandering.

During preliminary observations, we encountered both age groups of cleaners pecking on *Isopora palifera* (Figure 1c; Video S1, morphological identification, formerly *Acropora palifera*). We recorded whether a cleaner was observed pecking on *I. palifera* and how much time it spent associating with this coral (swimming within 5 cm, Figure 1c). Cleaners were not observed frequently pecking at any other coral species. Again, this pecking behavior was separated by whether the cleaner was at their station or wandering across the reef. When quantifying cleaning station characteristics (see above) one observer also estimated (to the nearest 5%) the percentage cover of *I. palifera* within each station. We also observed and recorded any instances of intraspecific cleaning between individuals and any substrate flashing. Flashing involves a fish swimming toward an inanimate object with a curved body position and contacting the object with the convex portion of their body (Video S2). Like cleaning, flashing links to hygiene regulation and, in particular, the dislodging of ectoparasites (Wyman & Walterswyman, 1985).

### 2.4 Data analysis

All analyses were carried out in R, version 3.4.3 (R Core Team, 2017). For analyses, we used a combination of general linear models (GLM), generalized linear mixed models (GLMM, package “lme4”; Bates, Maechler, Bolker, & Walker, 2015) and generalized additive models for location, scale and shape (GAMLSS, package “gamlss”; Rigby & Stasinopoulos, 2005, see Table 1). GAMLSS models replaced overdispersed GLMMs. Model assumptions and fits as specified by Bolker et al. (2009) were assessed using residual plots, and all continuous predictors were scaled and centered around zero to facilitate model convergence. Best fitting model selection was based on Akaike information criterion (AIC) using a backward elimination approach (with delta < 2). The significance of main effects was determined by comparing models with and without the main effect. Since repeated observations were carried out on the same cleaning stations across the study, “StationID” was included as a random term in all behavioral models (Table 1).

**Table 1:** Model structures used to investigate the cleaning dynamics of juvenile and adult bluestreak cleaner wrasse (*Labroides dimidiatus*) occupying a tidal lagoon on the Great Barrier Reef

Question	Model	Type	Family
Do station characteristics differ between juveniles and adults?	**Station volume** ~ Age	GLM	Gamma, identity link
	**Percentage cover** ~ Age	GLM	Inverse Gaussian, identity link
***Behavioral models***	All models below contain: + Observer ID + Time of day*Tide height + (1|StationID)		
Do adults spend more time wandering on the reef than juveniles?	**Wander time/Time spent in view** ~ Age	GAMLSS	Beta inflated
Do cleaning behaviors differ between wandering versus occupying a station (location type)?	**Clean frequency/Time spent at location type** ~ Location type*Age + Proportion of observation spent on own + (1|ObservationID)	GAMLSS	Beta binomial
	**Clean duration/Time spent at location type** ~ Location type*Age + Proportion of observation spent on own + (1|ObservationID)	GAMLSS	Beta binomial
	**Noncleaning interaction frequencies/Time at location type ~ **Location type*Age + Proportion of observation spent on own + (1|ObservationID)	GAMLSS	Beta binomial
	**Bite tendency ~ **Location type*Age + Proportion of observation spent on own + offset(Time at location type^i^) + (1|ObservationID)	GLMM	Binomial, logit link
Do juveniles and adults differ in their interactions with *Isopora palifera?&* Do *Isopora palifera* interactions link with cleaning behavior?	**Time spent associating with *I. palifera*/Time spent in view ~ **Age+Age/(Clean frequency + Clean duration + Noncleaning interaction frequencies + Proportion of observation spent wandering + Bite tendency) + Proportion of observation spent on own + offset(Time spent in view^ii^)	GAMLSS	Beta binomial
	***I. palifera* peck tendency** * ~ *Age+Age/(Clean frequency + Clean duration + Noncleaning interaction frequencies + Proportion of observation spent wandering + Bite tendency) + Proportion of observation spent on own + offset(Time spent in view^ii^)	GLMM	Binomial, probit link

Note

A combination of general linear models (GLM), generalized additive models for location, scale and shape (GAMLSS) and generalized linear mixed models (GLMM) was used. * denotes interaction term while (1|variable) shows factors specified as random terms to account for repeated observations. Offset accounts for variable observation lengths as a result of cleaner moving in and out of view for binary dependent variables: ^i^ = rescaled from one to 10 and log transformed to aid model fit. ^ii^ = rescaled from 0.1 to one and log transformed.

Data were collected by three observers so “ObserverID” was included as a fixed effect in all behavioral models (Table 1). Where there was significant variation between observers, which could have simply represented the natural variation in cleaning behavior (since all observed all cleaning stations), we reported the significance of fixed effects with and without the inclusion of “ObserverID” only if the result became nonsignificant (*n* = 2 cases, “cheating” bite tendency and *I. palifera* association time). This determined whether results were still consistent even when excluding this source of variation. Time of day and tide height can influence the behavior of many reef fish (Eggertsen, Hammar, & Gullström, 2016; Grutter, 1996); thus, both were included as an interaction term in all behavioral models (Table 1). For each observation, mean tide heights were calculated using “TideHarmonics” (Stephenson, 2016), based on hourly tide predictions for One Tree Island (from: Australia's Bureau of Meteorology). Any tide heights that were predicted to be less than 1.52 m by “TideHarmonics” were recoded to 1.52 m as this represents the lowest tide for the lagoon (Ludington, 1979). To account for potential effects of other bluestreak wrasse on observed behaviors, we also included the proportion of time a cleaner spent alone (i.e., no other cleaners present) as a fixed effect (Table 1).

During focal observations, fish could be lost temporarily (cleaner out of view) and/or permanently, before observations ended, meaning that observation lengths varied (mean ± *SE* observation length = 1,062.51 ± 22.82 s)—all behavioral models thus accounted for variable observation lengths (Table 1). For frequency/duration models (Table 1), variable observation times were accounted for by weighting values, while for binary dependent variables relating to tendency (Table 1), observation times were specified as an offset. Prior to this offset, observation time was rescaled (Table 1) using the scales package (Wickham, 2017). This method does not remove the variability between values, but simply transforms data to aid model fit.

Firstly, a GAMLSS model determined whether adults and juveniles differed in the proportion of time spent wandering across the reef (Table 1). For this beta inflated model (Table 1), “nu,” “tau,” and “sigma” parameters were also specified using stepGAIC call to improve model fit. Using two separate GLMs (Table 1), we then determined whether cleaning station (*n* = 19) ellipsoid volumes and *I. palifera* percentage covers differed between adults and juveniles. Given that percentage covers were estimated to the nearest 5% (except to the nearest 1% when < 5%, *n* = 1 case), data could thus be considered as ordinal. As such, percentage cover data were rescaled from one to 10. Rescaled percentage covers were square‐root transformed prior to analysis. Other methods for analyzing percentage data (e.g., binomial model, logit transformation) produced poor fitting models (assessed using residual plots).

To test whether cleaning behaviors differed between wandering versus occupying a station, we first separated cleaning frequencies, durations, noncleaning interaction frequencies, and bite tendency (0 = not observed, 1 = observed) into where it occurred on the reef for each observation. This meant that “ObservationID” was now included along with “StationID” as random terms, due to repeated measures within observations and across stations (Table 1). We used four separate models (Table 1) to determine whether cleaning behaviors differed between adults and juveniles. For the model investigating differences in bite tendency, we also included respective cleaning frequencies and durations as fixed effects (Table 1) to determine whether biting (“cheating”) likelihood increases with reduced cleaning.

As we observed both adult and juvenile bluestreak wrasse associating with, and pecking at, *I. palifera,* we finally determined whether juveniles and adults differed in their interactions (association time and peck tendency) with *I. palifera* and whether these interactions related to differences in cleaning behaviors (Table 1): cleaning behaviors were hence nested within “Age” (Table 1). For each observation, we calculated the time the cleaner spent associating with *I. palifera* and assigned observations as to whether individuals were observed pecking at *I. palifera* or not.

## 3 RESULTS

### 3.1 Cleaner habitat use in a tidal environment

**Figure 2:**
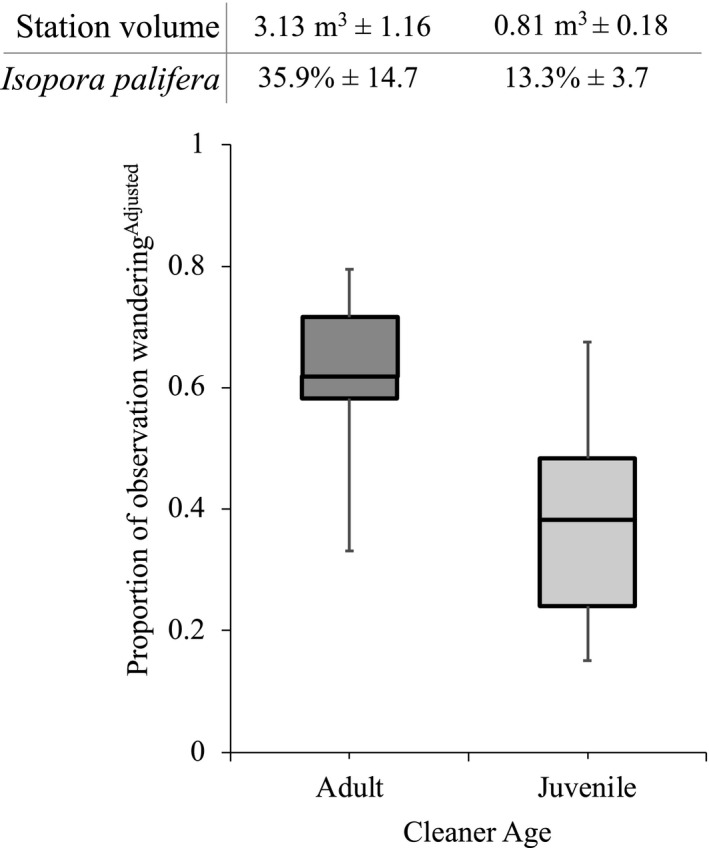
Adult *Labroides dimidiatus* spent more time wandering from their cleaning stations than juveniles on One Tree Reef, Australia. Boxplot presents median and inter‐quartile ranges of response values from GAMLSS model, along with maximum and minimum proportions. For raw unadjusted value figures see Supplementary materials. Station volume represents the mean ± *SE* station ellipsoid volume of adults versus juvenile stations, while *Isopora palifera* shows mean ± *SE* estimated percentage *I. palifera* coverage on stations

Compared to juveniles, adult bluestreak wrasse (*L. dimidiatus*) wandered more frequently across the reef, spending over 50% of their time wandering away from their stations (Figure 2; GAMLSS, *χ* ^2^_1_ = 6.91, *p* < .001, _model_ *R* ^2^ = 62.4%). Despite this increased wandering, adults also occupied larger cleaning stations than juveniles (Figure 2; station volume GLM, *F*_1,17_ = 8.53, *p* = .010, _model_ *R* ^2^ = 33.4%, *I. palifera* percentage GLM, F_1,17_ = 4.36, *p* = .052, _model_ *R* ^2^ = 20.4%).

### 3.2 Cleaning behavior in tidal environment

**Figure 3:**
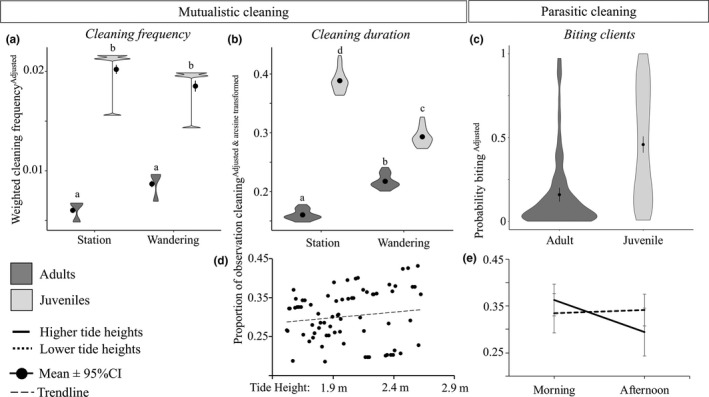
Mutualistic and parasitic cleaning behaviors of juveniles versus adult bluestreak wrasse (*Labroides dimidiatus*) wandering across the reef or occupying cleaning stations in a tidal lagoon. The shape of the violin plots represents the observed range of (a) cleaning frequencies, (b) cleaning durations, and (c) biting “cheating” likelihood (adjusted response values from GAMLSS models and a GLMM), while shape thickness shows how frequently these data values occurred. Point and lines show mean ± 95% CI. Cleaning duration data are arcsine transformed for figure clarity. Letters represent significance groupings based on Tukey's test and *p* < .05. (d) Shows the trend between tide height and time spent cleaning (*p* = .050), while (e) represents the significant interaction between mean tide height and time of day and shows their effect on wrasse biting probability (error bars show standard error around the mean value). For analyses, tide height and time of day were considered as continuous predictors but are presented categorically here to show effect. Low tide represents heights of 1.52–1.9 m while high tides consider heights between 1.91 and 2.63 m (data were split to create equal groups). For raw unadjusted value figures see Supplementary materials

We observed 2,803 cleaning interactions across 52 hr 49 min and 50 s of observations. Juveniles wrasse cleaned more frequently and spent more time cleaning than adults (Figure 3). Within age groups, juveniles spent more time cleaning when at their stations, while adults spent more time cleaning when wandering (Figure 3; frequency GAMLSS, *χ* ^2^_1_ = 4.03, *p* = .045, _model_ *R* ^2^ = 30.5%, duration GAMLSS, *χ* ^2^_1_ = 23.07, *p* < .001, _model_ *R* ^2^ = 37.0%). Wrasse also tended to spend more time cleaning at higher tides (Figure 3; GAMLSS, *β* = 0.11, *χ* ^2^_1_ = 3.76, *p* = .052).

In addition to cleaning, we observed 2,717 interactions which involved a cleaner inspecting or chasing a client. These inspection/food searching interactions did not result in cleaning and were more frequently observed when the cleaner was wandering compared to when occupying its station (GAMLSS, *χ* ^2^_1_ = 8.92, *p* = .003, _model_ *R* ^2^ = 8.07%, mean inspection frequency ± *SE* (weighted by observation length, s), station = 0.016 ± 0.002, wandering = 0.019 ± 0.002).

Cleaners were more likely to bite (cheat) clients when they cleaned more frequently and when they cleaned clients for shorter durations (GLMM, frequency: *β* = 1.53, *χ* ^2^_1_ = 32.75, *p* < .001, duration: *χ* ^2^_1_ = 6.54, *p* = .011, _model_ *R* ^2^ = 64.2%). Adults and juveniles also differed in their probability of cheating; juveniles were more likely to cheat clients (Figure 3; GLMM, *χ* ^2^_1_ = 10.45, *p* = .001), and there was a tendency for juveniles to cheat clients when at their stations compared to adults occupying stations (GLMM, *χ* ^2^_1_ = 3.19, *p* = .074, *p* = .096 without including significant observer effect). Cheating probability did not depend on tide in the earlier hours of the day, while later in the day, wrasse were more likely to bite at lower tides (Figure 3; GLMM, *χ* ^2^_1_ = 4.57, *p* = .032, *p* = .096 without including significant observer effect).

### 3.3 A reduced reliance on cleaning behavior?

**Figure 4:**
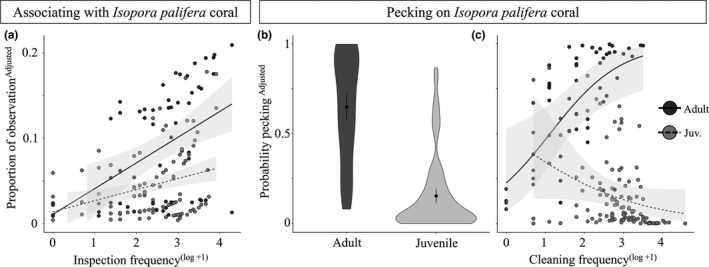
Adult and juvenile *Labroides dimidiatus* associated with and pecked on the coral *Isopora palifera* within a tidal lagoon. (a) Shows relationship between the proportion of time adults (solid line) and juveniles (dotted line) spent associating with *I. palifera* and their noncleaning inspection frequencies (adjusted response values shown from GAMLSS): a linear smoothing term was specified to show the relationship between *I. palifera* association time and inspection frequencies (±*SE*). The shape of the violin plot shows the distribution of adjusted model response values for (b) the pecking probability on *I. palifera* (GLMM), for juveniles and adults. Shape thickness represents the frequency of these data points occurring. Point and lines show mean ± 95% CI. (c) Shows the likelihood of *I. palifera* pecking, separated by age, negatively correlating with cleaning frequencies for juveniles (adjusted response values shown from GAMLSS). A binomial smoothing term was specified to show the relationship between pecking and cleaning frequency (adults = solid line, juveniles = dotted line ± *SE*). Inspection and cleaning frequencies were log (+1) transformed for clarity. For raw unadjusted value figures see Supplementary materials

Cleaners were also observed interacting with *I. palifera* coral: cleaners associated with, inspected, and/or pecked at, the coral branches (Video S1). Juveniles spent more time associating with *I. palifera* when they inspected clients (without cleaning) more frequently (Figure 4; GAMLSS, inspection frequency: *χ* ^2^_2_ = 10.06, *p* = .007, *p* = .085 without including significant observer effect, cheating tendency: *β* = −0.95, *χ* ^2^_2_ = 6.76, *p* = .034 but *p* > .100 without including significant observer effect, _model_ *R* ^2^ = 37.6%). Coral association was unaffected by cleaning behavior (frequency and duration) and reef location (wandering vs. station, GAMLSS, all *p* > .100). Cleaners that spent more time alone spent more time associating with *I. palifera* (GAMLSS, *β* = 0.30, *χ* ^2^_1_ = 5.40, *p* = .020).

*Isopora palifera* pecking was observed in 43% of observations, and adults were the most likely to coral peck (Figure 4; GLMM, *χ* ^2^_1_ = 15.99, *p* < .001, _model_ *R* ^2^ = 70.6%). Juveniles which cleaned less frequently were also more likely to coral peck (Figure 4; GLMM, *β* = −0.69, *χ* ^2^_2_ = 7.88, *p* = .020). *Isopora palifera* pecking was more likely to be observed in the morning (GLMM, *β* = −0.35, *χ* ^2^_1_ = 4.27, *p* = .039).

We also observed intraspecific wrasse cleaning on 19 occasions (observed in 5.0% of observations) and substrate flashing on 92 occasions (observed in 13.4% of observations). Compared to juveniles, adults intraspecifically cleaned and flashed the most frequently (number of observations intraspecific cleaning seen: adults = 8, juveniles = 1, number of observations flashing seen, adults = 18, juveniles = 6).

## 4 DISCUSSION

Here for the first time, we demonstrate the shift from a dedicated cleaning mutualism to a more facultative interaction for a model cleaner species, the bluestreak wrasse (*L. dimidiatus*) occurring in a tidal environment. Rather than relying solely on cleaning for nutrition, adult and juvenile wrasse were observed pecking at the common coral *I. palifera,* with juveniles decreasing their cleaning frequencies with coral pecking. Adult wrasse also spent more time wandering across the reef, rather than occupying fixed cleaning stations, compared to juveniles. While wandering, adults spent more time cleaning compared to when at their stations. Client biting (cheating) was frequently observed, with biting likelihood increasing with cleaning frequency and decreased cleaning duration. Juveniles were more likely to bite clients compared to adults. Within this environment, this “dedicated” cleaner species has, at some point, partly abandoned cleaning as a food acquisition strategy.

Bluestreak cleaner wrasse is a model species for cleaning studies; their cleaning behavior has been the focus of hundreds of studies dating back to at least the 1950s (e.g., Randall, 1958, reviews: Feder, 1966; Losey, Grutter, Rosenquist, Mahon, & Zamzow, 1999; Côté & Soares, 2011; Vaughan et al., 2017). Importantly, across these studies, in situ bluestreak wrasse have consistently been reported to rely solely on client‐gleaned material for nutrition (Côté & Soares, 2011; Vaughan et al., 2017, with the exception of plankton feeding documented by Losey, 1972). To our knowledge, bluestreak wrasse have not been documented to abandon cleaning (although they can feed opportunistically on alternatively offered food rewards; Grutter, 1996), even under home aquarium conditions (instead they do not acclimatize well and have high mortality; Wabnitz, 2003), but here for the first time, we observed this iconic cleaner also pecking on the coral *I. palifera.* Given that coral pecking likelihood increased with a reduced cleaning frequency for juveniles, and was more likely in the morning (cleaning can also be more frequent in the morning; Grutter, 1996), we suggest that wrasse may be gaining a food source from the coral, with the behavior displacing cleaning for juveniles and supplementing feeding for adults (contrasting supplemental juvenile *Thalassoma bifasciatum* cleaning; Dunkley et al., 2018). Many reef species are observed feeding on *I. palifera* (e.g., Labridae; Cole, 2009 and Chaetodontidae; Nagelkerken, Velde, Wartenbergh, Nugues, & Pratchett, 2009), but what the acquired food source is (e.g., coral mucus, polyps, zoo‐/phytoplankton and/or periphyton) remains unknown: bluestreak wrasse have been reported to consume naturally occurring and artificially placed zooplankton in situ (Grutter, 1996; Losey, 1972). Cleaners did appear, however, to be selectively picking material from the coral, since inspection behavior can be observed in the [Link], [Link] footage: cleaners may thus be searching for a particular type of food source that is not uniformly distributed across the coral (similar to client inspection behavior in juvenile *T. bifasciatum* cleaning; Dunkley et al., 2018). Cleaner wrasse coral pecking was more prevalent in adults and begins to mirror the ontogenetic shift in cleaning behavior of some facultative cleaners: juveniles clean, while adults become more generalist and consume other food types (e.g., feeding on coral polyps by *Labrichthys unilineatus* and *Diproctacanthus xanthurus*, see Cole, 2009, Grutter & Feeney, 2016, and sponge‐feeding by *Pomacanthus paru*, see Hourigan, Stanton, Motta, Kelley, & Carlson, 1989). Ectoparasite consumption is thought to be less nutritionally beneficial than consuming other material (e.g., sponge; White et al., 2007, or mucus; Eckes, Dove, Siebeck, & Grutter, 2015) and daily environmental fluctuations in this shallow tidal study site may mean that dedicated cleaning is no longer a beneficial food acquisition strategy: increased temperatures combined with low oxygen will physiologically increase metabolic demands (Clarke & Fraser, 2004; Holt & Jørgensen, 2015). Indeed, Paula et al. (2019) also documented a reduction in cleaning interactions under altered physiological laboratory conditions but did not offer alternative food resources—this would make an interesting further study on cleaning breakdown to supplement this single site observation.

In addition, within tidal lagoons, the movement of fish species and their larvae is tidally limited (Thresher, 1983) consequently restricting ectoparasite abundance and diversity (Grutter, 1994). Thus, here, cleaning interactions at lower tides may represent repeated interactions between the same few individuals: this may result in a reduction of ectoparasite abundances, and hence food rewards on client fish, as repeat interactions increase (Cheney & Côté, 2003). This may explain why adult wrasse spent more time cleaning when wandering and at higher tides—to capitalize on restricted client movement. Ultimately, environmental variability in client abundance and diversity may promote cleaners to search for food and switch their foraging strategy. Interestingly, many fish species we regularly observed as clients (e.g., *Chaetodon rainfordi*, *Lutjanus carponotatus* and *Scolopsis bilineatus*) are considered resident reef members and are not expected to move off the reef during tide changes—perhaps ensuring a more stable source of food for the cleaners. Data on the abundance and diversity of available client species in relation to cleaning patterns would thus be useful for future study.

Within this tidal lagoon, the switch toward a more facultative cleaning strategy suggests that dedicated cleaning has, at some point, broken down: longitudinal studies of cleaning are thus more important than ever. A reduction in cleaning, however, may have its own costs, perhaps constraining the extent to which the interaction is abandoned. Part‐time cleaners often visit cleaning stations or attempt to elicit cleaning from heterospecifics (Arnal, Côté, Sasal, & Morand, 2000; Dunkley et al., 2018, 2019; Sazima, Moura, & Sazima, 1999), and here, we documented both intraspecific cleaning between adult bluestreak wrasse (only previously documented once between a cleaner pair; Clague, Newport, & Grutter, 2011) and substrate flashing (to our knowledge, not previously documented for this species in situ, but ex situ documentation by Kohda et al., 2018). These behaviors will all help to control an individual cleaner's own parasite loads: A reduction in client cleaning may increase cleaners own susceptibility to parasites and disease. In addition, a restriction in fish movements may reduce the host to parasite ratio on the reef, meaning cleaners themselves may be vulnerable to higher parasite loads.

In this study, we also quantified for the first time, a novel change in bluestreak cleaning behavior. Animals can adapt their habitat use as a rapid response to a changing environment (Van Buskirk, 2012), and here, bluestreak wrasse (predominantly adults) wandered across the reef (as anecdotally described in Wilson et al., 2014) rather than spending all their time at their fixed cleaning stations waiting for clients (e.g., Bshary & Schäffer, 2002; Côté & Soares, 2011). Wanderers inspected/chased clients more frequently and spent more time cleaning, compared to when at their station. Bicolor cleaner wrasse (*Labroides bicolor*) wander to find clients (Mills & Côté, 2010; Oates et al., 2010), and we suggest that this wandering also represents a food searching behavior for bluestreaks. In this tidally restricted environment, adults, which will have higher nutritional requirements than juveniles (bigger bodies require higher energy demands; Speakman, 2005), adopt a riskier foraging behavior by searching for food themselves: usually in nontidal environments, waiting at cleaning stations increases cleaning gains compared to wandering (bluestreak vs. bicolor wrasse comparison; Oates et al., 2010). Ultimately, this wandering behavior may maintain the occurrence of the mutualism.

Mutualists can increase the magnitude of their rewards by exploiting the system (Sachs & Simms, 2006), and here, we also observed juveniles cheating (biting more calorific mucus from clients; Eckes et al., 2015; Wilson et al., 2014) more frequently than adults; an intriguing result not usually observed (contrasts Mills & Côté, 2010). This parasitism may help juveniles achieve their nutritional demands in this tidally restricted environment (juveniles wandered less than adults). Bluestreak wrasse usually alter their mutualistic versus parasitic cleaning behavior to ensure the mutualism is maintained (Binning et al., 2017; Bshary, 2002) but here, although we did document increased biting tendency with increased cleaning frequency, this mutualism may be shifting to a more parasitic interaction.

Overall, we found for the first time, that under tidal conditions, both adults and juvenile bluestreak cleaner wrasse were part‐time facultative cleaners, by also pecking on the coral *I. palifera*. We thus found that cleaners partly abandon the mutualism, showing a reduced reliance on cleaning as a food source. Juveniles also appear to exploit the system by parasitizing clients more frequently than adults. The mutualism however is only partly abandoned, and adults show novel plasticity in their foraging behavior by wandering across the reef searching for food, rather than waiting at their cleaning stations for clients. Perhaps as a result of this adaptation, cleaners were also observed to intraspecifically clean and flash their bodies on rocky substrate. Thus, there still must be some benefits acquired through cleaning, or constrictions that prevents this cleaner from switching completely to an alternative feeding method (which has been observed for other mutualists; Sachs & Simms, 2006). What these constraints are, however, is unknown. Through their large number of species interactions, and removal of parasites, dedicated and part‐time facultative cleaner species play an important role in the ecological community structure (Floeter, Vazquez, & Grutter, 2007; Quimbayo et al., 2018). More cleaner species adopt part‐time rather than dedicated cleaning strategies (Vaughan et al., 2017), and since a dedicated cleaner showed a reduced cleaning reliance in this tidal lagoon, it begs the question how stable and pervasive facultative cleaning strategies will be under locally fluctuating environmental conditions.

## CONFLICT OF INTEREST

We have no competing interests.

## AUTHOR CONTRIBUTIONS

K.D., A.J.W., S.E.P, and J.C. conceived the study; K.D., S.E.P., and J.C. collected the data; K.D. conducted the statistical analyses and drafted the manuscript; all authors contributed to manuscript revisions.

## Data Availability

Analyses reported in this article can be reproduced using the data and R‐script made available by Dunkley, Ward, Perkins, and Cable (2020).
