# Supplementary material for: To clean or not to clean: Cleaning mutualism breakdown in a tidal environment
Source: Ecol Evol. 2020 Feb 28;10(6):3043–54. doi: 10.1002/ece3.6120 (PMC7083704; doi:10.1002/ece3.6120)
Supplement: Supplementary file 1 [file ECE3-10-3043-s001.pdf]

TO CLEAN OR NOT TO CLEAN: CLEANING MUTUALISM BREAKDOWN IN A TIDAL ENVIRONMENT: SUPPLEMENTARY MATERIALS

Station volume	3.13m^3 ± 1.16	0.81m^3 ± 0.18
Isopora palifera	35.9% ± 14.7	13.3% ± 3.7

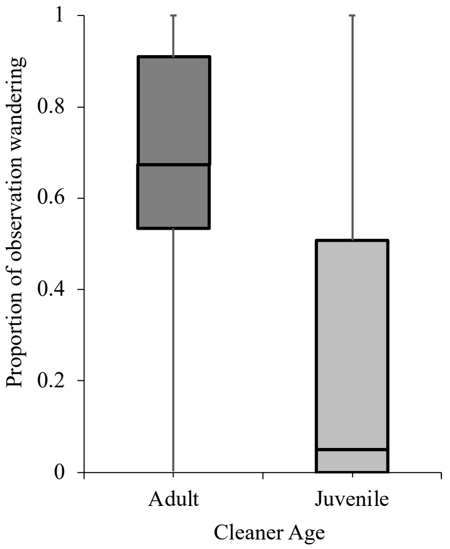

Figure 2: Adult *Labroides dimidiatus* spent more time wandering from their cleaning stations than juveniles on One Tree Reef, Australia. Boxplot presents median and inter-quartile ranges of raw values, along with maximum and minimum proportions. Station volume represents the mean ± S.E. station ellipsoid volume of adults versus juvenile stations, whilst *Isopora palifera* shows mean ± S.E. estimated percentage *I. palifera* coverage on stations.

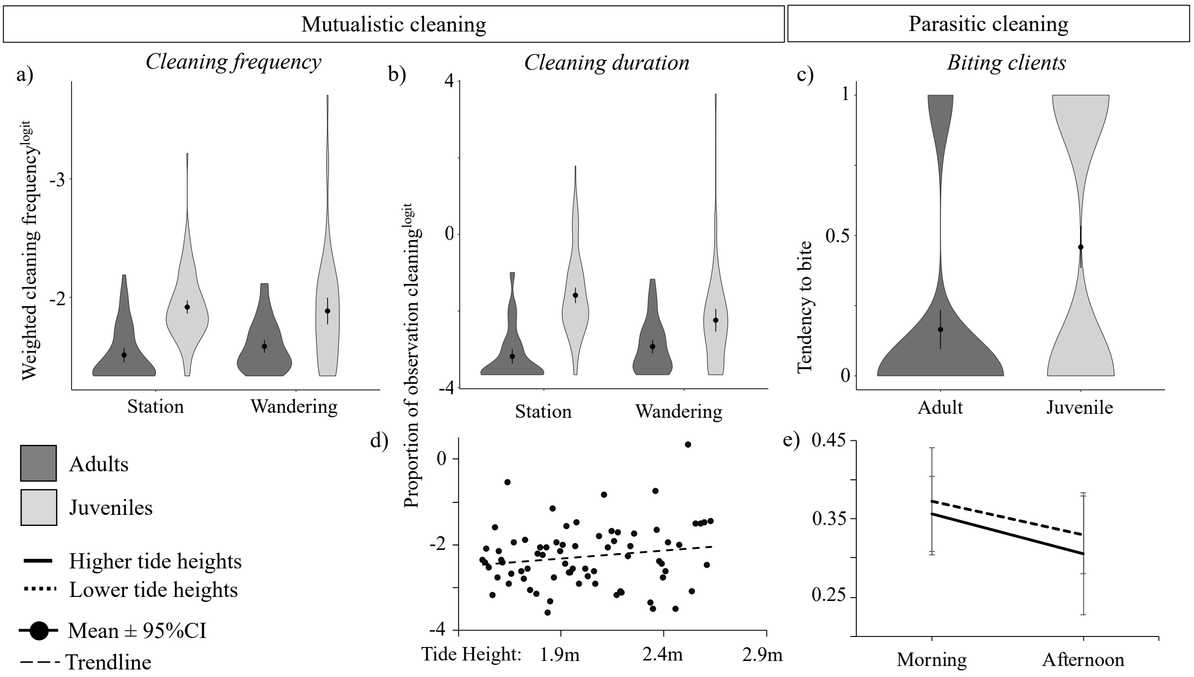

Figure 3: Mutualistic and parasitic cleaning behaviors of juveniles versus adult bluestreak wrasse (*Labroides dimidiatus*) wandering across the reef or occupying cleaning stations in a tidal lagoon. The shape of the violin plots represents the observed range of a) cleaning frequencies, b) cleaning durations and c) biting 'cheating' likelihood (raw values, cleaning

frequency and duration adjusted by observation time), whilst shape thickness shows how frequently these data values occurred. Point and lines show mean ± 95 % CI. Cleaning frequency and duration data is logit/arcsine transformed for figure clarity. Letters represent significance groupings based on Tukey's test and $p < 0.05$. d) Shows the trend between tide height and time spent cleaning ($p = 0.050$), whilst e) represents the significant interaction between mean tide height and time of day and shows their effect on wrasse biting probability (error bars show standard error around the mean value). For analyses, tide height and time of day were considered as continuous predictors but are presented categorically here to show effect. Low tide represents heights of 1.52 m – 1.9 m whilst high tides consider heights between 1.91 m and 2.63 m (data were split to create equal groups).

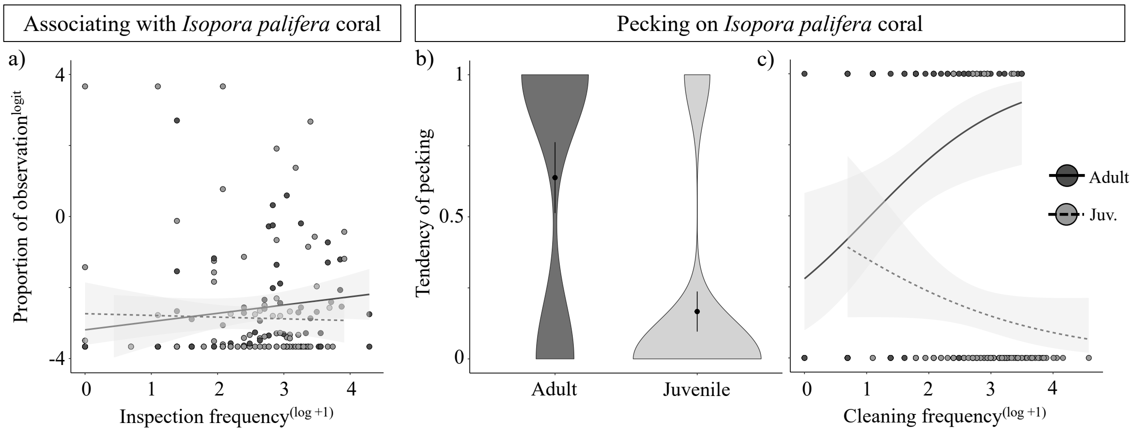

Figure 4: Adult and juvenile *Labroides dimidiatus* associated with and pecked on the coral *Isopora palifera* within a tidal lagoon. (a) Shows relationship between the proportion of time adults (solid line) and juveniles (dotted line) spent associating with *I. palifera* and their non-cleaning inspection frequencies (logit transformed for clarity): a linear smoothing term was specified to show the relationship between *I. palifera* association time and inspection frequencies (± S.E.). The shape of the violin plot shows the distribution binary values for (b) the pecking probability on *I. palifera*, for juveniles and adults. Shape thickness represents the frequency of these data points occurring. Point and lines show mean ± 95% CI. c) Shows the likelihood of *I. palifera* pecking, separated by Age, negatively correlating with cleaning frequencies for juveniles. A binomial smoothing term was specified to show the relationship between pecking and cleaning frequency (adults = solid line, juveniles = dotted line ± S.E). Inspection and cleaning frequencies were log (+1) transformed for clarity.
